# *In Vivo* Non-Invasive Tracking of Macrophage Recruitment to Experimental Stroke

**DOI:** 10.1371/journal.pone.0156626

**Published:** 2016-06-24

**Authors:** Marion Selt, Annette Tennstaedt, Andreas Beyrau, Melanie Nelles, Gabriele Schneider, Clemens Löwik, Mathias Hoehn

**Affiliations:** 1 In-vivo-NMR Laboratory, Max Planck Institute for Metabolism Research, Cologne, Germany; 2 Dept. of Radiology, Leiden University Medical Center, Leiden, The Netherlands; 3 Percuros B.V., Enschede, The Netherlands; Johns Hopkins University, UNITED STATES

## Abstract

Brain-infiltrating monocyte-derived macrophages are one of the key players in the local immune response after stroke. It is now widely accepted that the inflammatory response is not an exclusively destructive process. However, the underlying molecular mechanisms needed for proper regulation still remain to be elucidated. Here, we propose an *in vitro* labelling strategy for multimodal *in vivo* observation of macrophage dynamics distinguished from brain-residing microglia response. Prior to intracerebral transplantation into the striatum of recipient mice or systemic administration, monocytes and macrophages, isolated from luciferase-expressing mice, were labelled with superparamagnetic iron oxide particles. Temporo-spatial localization was monitored by magnetic resonance imaging, whereas survival of grafted cells was investigated using bioluminescence imaging. The labelling procedure of the isolated cells did not significantly influence cell characteristics and resulted in detection of as few as 500 labelled cells *in vivo*. Two weeks after stereotactic transplantation, the luciferase signal was sustained traceable, with approximately 18% of the original luciferase signal detectable for monocytes and about 30% for macrophages. Hypointensity in MRI of the graft appeared unaltered in spatial location. In a therapeutically relevant approach, systemic cell administration after stroke resulted in accumulation mostly in thoracic regions, as could be visualized with BLI. For detection of homing to ischemic brain tissue more cells need to be administered. Nevertheless, during parallel MRI sessions recruitment of i.v. injected cells to the lesion site could be detected by day 2 post stroke as scattered hypointense signal voids. With further increase in sensitivity, our multi-facetted labelling strategy will provide the basis for *in vivo* tracking and fate specification of tissue-infiltrating macrophages and their distinct role in stroke-related neuro-inflammation.

## Introduction

Ischemic or traumatic brain injuries or other cerebral diseases are accompanied by a strong local inflammatory response in the affected tissue [1, 2]. Main key players are the CNS-resident microglia and the blood-borne CNS-infiltrating monocyte-derived macrophages (in the following called MΦ). It is commonly accepted that brain inflammation contributes to pathogenesis in acute as well as chronic neurodegenerative diseases. Recent findings also highlight the importance for neuroprotection, axonal regeneration and cell recovery [3–7], which display the positive potential of inflammatory processes. The challenge is that the response is not well controlled and therefore can turn out inhibitory to recovery [8]. Therefore, better understanding of the cross-talk between the brain and the immune system is of great importance in order to find new therapeutic approaches.

Upon brain injury CNS-resident microglia are rapidly activated. Besides diverse functions like removing dead cells and cell debris, they initiate the formation of the glial scar [9]. Secretion of pro-inflammatory mediators induces the infiltration of circulating monocytes to the lesion site [10]. It has been suggested that the monocytes play a regulatory role in the inflammation process [4–7, 11, 12] with the essential function of co-localization with the glial scar. This step is crucial for proper scar resolution and termination of the local microglial response.

In order to investigate the role of infiltrating MΦ in the stroke-related inflammatory response distinct from that of microglia, one has to overcome one major obstacle. As activated brain residing microglia and tissue infiltrating MΦ are indistinguishable by standard histological or immunohistological techniques [7], the use of transgenic animal models is highly desirable. A reliable and powerful *in vitro* labelling protocol with contrast agents for magnetic resonance imaging (MRI) combined with transgenic donor animals enables a versatile observation of the fate and temporal as well as spatial localization of transplanted transgenic monocytes and MΦ *in vivo*. MRI permits to characterize ischaemic brain lesions. Well-established protocols combined with the use of contrast agents (superparamagnetic particles of iron oxide–SPIOs) allow for investigation of the spatial localization of implanted cells in the host brain tissue [13, 14]. However, SPIO-based MRI does not provide information on cell viability. This has to be achieved by combining physical SPIO labels with transgenic cells expressing the luciferase gene, as previously shown [15–18]. Firefly luciferase oxidates the substrate D-Luciferin in an energy dependent chemical reaction, in which photons are generated. This reaction requires the presence of ATP and oxygen. Therefore the emitted bioluminescence signal can be used for cell localization and serves as an indicator for cell viability.

In the present study we I) carefully assessed optimal labelling strategies with SPIO particles for minimal or no label influence on monocytes and MΦ *in vitro*; II) investigated labelling effectiveness and detection sensitivity *in vitro* and *in vivo*; III) determined longevity of contrast agent and spatial cell localization in a qualitative manner (SPIO-MRI); IV) monitored spatial cell localization longitudinally (SPIO-MRI) and assessed graft survival via bioluminescence imaging (BLI); and, finally, V) applied our findings to experimental stroke, in order to image the recruitment time profile and fate of systemically administered monocytes and MΦ *in vivo* (SPIO-MRI and BLI).

## Materials and Methods

### Animals

All animal experiments were conducted according to the guidelines laid out in the German Animal Welfare Act and approved by the local authorities (Landesamt für Natur, Umwelt und Verbraucherschutz Nordrhein-Westfalen). Animals were housed in individually ventilated cages at a 12/12 h light/dark cycle and were provided *ad libitum* with standard diet and water. For experiments with WT cells adult male C57BL/6 mice (20–35 g body weight) were used, whereas for experiments with transgenic cells (luc^+^) adult transgenic β-actin-luc mice (FVB background, males and females; 20–35 g) were used as cell donors. WT cells were transplanted into C57BL/6 recipient mice, for transgenic luc^+^ cells FVB-WT mice were used as recipients, in order to transfer the cells into organisms displaying the same genetic background. Transplanted animals underwent sequential BLI and MRI on days 1, 7 and 14 post transplantation. Animals with systemic administration (i.v.) of monocytes/MΦ were scanned directly after middle cerebral artery occlusion (MCAO) surgery, as well as on days 2, 3 and 4 post MCAO.

### Isolation of monocytes and generation of bone marrow (BM) derived MΦ

For monocyte isolation as well as differentiation of MΦ, BM cells were collected by flushing the shafts of tibia and femur with MACS buffer (Miltenyi Biotec, Bergisch Gladbach, Germany) or PBS (Gibco, Life Technologies, Paisley, UK), respectively, using 26G needles. Monocytes were isolated from BM using the monocyte isolation kit (Miltenyi Biotec) following the manufacturer’s protocol.

For differentiation of MΦ, BM cells were incubated for 5 min with erythrocyte lysis buffer (RBC lysis buffer, multi species, eBioscience San Diego, USA) and filtrated through a 70 μm BD-filter (BD biosciences, USA). After centrifugation (300 x g for 5 min at 4°C) 5x10^6^ cells were plated on petri dishes (10 cm Ø, Greiner Bio-One, Frickenhausen, Germany) in 10 ml RPMI 1640 medium (+Glutamax; Gibco, Life Technologies, Paisley, UK), substituted with 10% FBS, 1% Penicillin/Streptomycin, 1 mM sodium pyruvate, as well as 50 ng/ml M-CSF (R & D Systems, Minneapolis, USA). On day 4 of differentiation, M-CSF was added within 1 ml of fresh medium to a final concentration of 50 ng/ml. On day 7, cells were detached with StemPro accutase (Gibco, Life Technologies, Paisley, UK), counted with trypan blue (Life Technologies) to determine viable/dead ratio, and plated as desired.

### Labelling cells with MRI contrast agent

Monocytes/MΦ were seeded at 5x10^5^ per 4-well plate (Nunc, Roskilde, Denmark) at least 1 h prior to contrast agent labelling. Immortalized J774A.1 MΦ (Banca Biologica e Cell Factory, San Martino, Genova, Italy) were seeded 1 day before contrast agent labelling at 5x10^5^ per 6-well plate (Greiner Bio-One, Frickenhausen, Germany) in 2 ml DMEM medium (+Glutamax, Gibco, Life Technologies, Paisley, UK), substituted with 10% FBS. Incubation of all cells with SPIO nanoparticles (Nanomag, 250 nm diameter, Micromod, Rostock, Germany) was performed overnight at concentrations of 0, 50, 168, 250 or 500 μg Fe/ml. Labelled as well as unlabelled (control) cells were harvested with accutase and centrifuged at 300 x g for 5 min. Viable cells were counted with trypan blue and subsequently prepared in the appropriate numbers in one of the following ways: I) dissolved in HBSS (Life Technologies) for transplantation (2 μl) or intravenous (i.v.) injection (200 μl), II) dissolved in PBS for photometric iron content analysis or phantom MRI, III) fixed with paraformaldehyde (PFA) for Prussian Blue (PB) staining, and IV) dissolved in MACS buffer for FACS analysis.

### Intracellular iron content analysis

Iron uptake capacity was quantitatively measured using a colorimetric ferrozine assay based on the procedure reported by Riemer et al. [19]. Briefly, cells were incubated overnight with different iron concentrations of Nanomag. Samples containing 9x10^4^ to 1x10^5^ cells were dissolved and incubated at room temperature for 2 h in sodium hydroxide solution (NaOH; 0.5M) to lyse the cells. 20 μl of the supernatant as well as the same volume of standard (ammonium iron (III) citrate) were mixed each with 20 μl of hydrogen chloride (HCl; 10 mM) and the same volume of iron releasing reagent [1:1 potassium permanganate (KMnO_4_; 4.5%); HCl (1.4 M)] and incubated at 60°C for 2 h in the shaker. After cooling-down, iron detection reagent was added to each sample and standard, followed by incubation for another 30 min. 50 μl of each sample were transferred into a well of a 96-well plate and absorbance was measured at 570 nm on a microplate reader.

### Live-cell viability assay

Luciferase-expressing (luc^+^) monocytes and MΦ were plated on black 96-well plates the day before measurement at 7.5x10^4^ cells within 200 μl medium supplemented with 10 ng/ml M-CSF. Cells were incubated overnight with contrast agent. After removing the contrast agent, cell viability was analysed *in vitro* after additional 2–3 h incubation at 37°C with the fluorescent redox indicator dye resazurin (Presto Blue, Thermo Fisher; 1:10 dilution in medium). Fluorescence was measured using a microplate reader at excitation wavelength 560 nm and an emission wavelength 600 nm. Alternatively, cell viability was recorded on the microplate reader using the bioluminescence signal intensity as the signal generation is ATP dependent. This approach could be applied both *in vitro* and *in vivo*.

### Phantom preparation

For *in vitro* MRI, appropriate numbers of control and SPIO labelled WT cells (1x10^3^, 5x10^3^, 1x10^4^, 5x10^4^) were dissolved in 30 μl PBS/low melting agarose (1%) mixture and transferred into a small agarose-filled PCR tube. Phantoms were positioned in an in-house built MRI holder, also filled with agarose to reduce susceptibility artefacts at the edges of the PCR tube.

### Anesthesia

All animals were initially anesthetized with 3–3.5% isoflurane in a 70:30 nitrous oxide/oxygen mixture. Throughout the experiments isoflurane was reduced to 1–2% for maintenance. During MRI measurements body temperature was continuously monitored with a rectal probe and maintained at 37.0 ± 1.0°C by adjusting the temperature of the warm water circulating through the animal cradle. Throughout surgical procedures, body temperature was monitored with a rectal probe and adjusted to above-mentioned temperature by an in-house built feedback-control system. During BLI measurements, animals were positioned on a heatable ground plate set to 37°C.

### Transplantation procedures

The cell transplantation protocol was adopted from previously described work [20]. Briefly, animals were anesthetized with isoflurane, received subcutaneous (s.c.) injection of 4 mg/kg Carprofen (Pfizer, Karlsruhe, Germany) for analgesia and remained fixed in a stereotactic frame (Stoelting, Dublin, Ireland) throughout surgery. Different numbers of cells in HBSS were injected into the brain in a total volume of 2 μl using a Hamilton syringe (26G needle; withdraw 1500 nl/min, injection 150 nl/min). For all injections the following coordinates relative to bregma were chosen using a stereotactic instrument (Stoelting): anterior-posterior (AP) +0.5; medial-lateral (ML) +2.0; dorso-ventral (DV) -3.0 for striatal injections into the right hemisphere. C57BL/6 WT animals got additional injections with the coordinates AP +0.5; ML -2.0; DV -3.0. For the *in vivo* MRI titration experiment, besides the two above mentioned coordinates, cells were additionally injected at: AP -1.0; ML +/-2.0; DV -3.0 as well as AP -2.5; ML +/-2.0; DV -3.0.

### Middle cerebral artery occlusion and systemic cell administration

For experimental stroke induction, animals were anesthetized with isoflurane and received an injection (s.c.) of 4 mg/kg Carprofen for analgesia. Blood flow was monitored via laser Doppler flowmetry on the *foramen ovale* of the skull. The *arteria carotis communis* was exposed and a silicon-coated filament was gently pushed through a small incision in the wall of the *arteria carotis communis* into the correct position on the bifurcation of the *arteria cerebri media (MCA)*. The animals were allowed to wake up during occlusion duration to reduce time under anesthesia. After 30 minutes occlusion of the MCA, animals were anesthetized again to remove the filament and to coagulate the incision in the artery. The animals were sutured, treated with 2 ml saline solution (NaCl s.c.) and allowed to recover. The following day, animals received either WT monocytes (labelled) or luc^+^ MΦ (labelled) or luc^+^ MΦ (control) via tail vein injection. Cell numbers ranged from 1x10^6^ to 6x10^6^ and were dissolved in a total volume of 200 μl HBSS.

### Magnetic resonance imaging

For acquisition of high-resolution magnetic resonance images, experiments were conducted on a horizontal 9.4T/ 20 cm or 11.7T/ 16 cm Biospec animal scanner system (Bruker BioSpin, Ettlingen, Germany) using ParaVision 5.1 software. Phantom MRI was conducted using a rat surface coil and resonator (both Bruker). *In vivo* MRI was carried out using a mouse quadrature surface coil for signal detection while radio frequency (RF) transmission was achieved with a quadrature resonator (both Bruker). After anesthesia, the mice were fixed in a standard animal holder (Bruker) with ear- as well as toothbars to prevent movement artefacts. Respiration rate was continuously monitored via DASYLab software (National Instruments, Austin, TX, USA) by placing a pressure sensitive pad under the animals’ thorax. Body core temperature was recorded with a rectal probe and manually adjusted via a warm water system for maintenance at 37.0 ± 1.0°C. The imaging protocol for *in vitro* MRI consisted of a multi-slice multi-spin-echo (MSME) sequence: 8 contiguous 0.5 mm thick slices, field of view (FOV) = 25x25 mm^2^, matrix = 256^2^, echo time/repetition time (TE/TR) = 11.25 ms/5,000 ms, 10 echoes, acquisition time (TA) = 16:00 min. The scan protocol for *in vivo* MRI consisted of a multi-gradient echo (MGE) sequence: either 6 contiguous 0.4 mm thin slices covering the cell graft (transplantation experiments), or 12 contiguous slices of the same thickness covering most of the ischemic lesion (systemic administration experiments), field of view (FOV) = 14x14 mm^2^, matrix = 128^2^, echo time/repetition time (TE/TR) = 3.5 ms/1,500 ms, 12 echoes, echo spacing = 5 ms, flip angle (FA) = 30°, acquisition time (TA) = 4:48 min; and MSME sequence: geometry and resolution identical to MGE, echo time/repetition time (TE/TR) = 10.25 ms/5,000 ms, 16 echoes, acquisition time (TA) = 10:40 min.

### Bioluminescence imaging

All bioluminescence imaging (BLI) experiments were performed using an IVIS Spectrum CT pre-clinical *in vivo* imaging system (PerkinElmer Life Sciences, Hopkinton, MA, USA). For *in vitro* imaging, luciferase-expressing cells were plated in different amounts (1x10^4^, 5x10^3^, 2.5x10^3^, 1.25x10^3^ and 625) in black 96-well plates (Corning Inc., Corning, NY, USA) and 100 μl PBS. 5 min after addition of 1 mM ᴅ-Luciferin (Synchem, Felsberg; Germany) images were acquired using an open filter and an auto-exposure-sequence of 1 min duration. The *in vivo* set-up included an open filter measurement with a binning of 8 and 13.2 cm field of view. Photon emission was recorded for 32 min (8 segments of 4 min duration) upon ᴅ-Luciferin injection (300 mg/kg; i.p.) according to a previously described optimized protocol [21]. BLI was recorded at different time points (1, 7 and 14 days) post transplantation of 7.5x10^4^ monocytes and MΦ (n = 3 for each cell type). In order to guarantee free photon emission, fur was removed on the animals’ heads. BLI signal change is expressed in % in relation to photon emission on day 1 after transplantation. Animals were allowed to recover from anesthesia for at least 3 h inbetween MRI and BLI measurements.

### Image postprocessing and data analysis

After MR image acquisition, all T2 and T2* maps were calculated on a voxel basis using a custom-made program written with IDL software (Version 6.4, Research Systems, Inc., Boulder, USA). For image visualization the generated maps were transferred to ImageJ software (Version 1.48g; National Institute of Health) and mean T2 and T2* relaxation times within selected regions of interest (ROIs) were calculated.

ROI analysis of the acquired BLI images was performed using Living Image software (Perkin-Elmer). Background noise is automatically subtracted by the camera. Hence, ROIs were positioned on the detected signals, as well as over the animal tail for calculation of the specific background signals arising from the animals.

### Flow cytometric analysis

Cells were detached from culture dishes using accutase, centrifuged at 300 x g for 5 min and counted. 10^5^ to 10^6^ cells were dissolved in MACS buffer and labelled with the following monoclonal antibodies (Table 1) according to the manufacturers’ protocols. Cells were analysed on a FACSAria III (BD Biosciences) system using FACSDiva software (Version 6.1.3). For both, luc^+^ monocytes and Mɸ, single cells were identified by forward scatter, excluding dead cells. Luc^+^ monocytes were gated for monocytic markers Ly6c and CD115. Luc^+^ Mɸ were gated for F4/80^+^/CD68^+^ or F4/80^+^/CD80^+^ populations, respectively. Positive populations were then further gated for CD86 and CD206, with CD115 and Ly6c as negative controls. Post-processing of the acquired FACS data was conducted using FlowJo Software (Version 10.0.8., Ashland, Oregon, United States) for histogram depiction of experiments.

**Table 1:** List of fluorochrome-coupled antibodies used for flow cytometry, with corresponding dilutions and information on supplier.

Antibody	Dilution	Supplier
PE-conjugated anti-CD115	1:10	Miltenyi
APC-conjugated anti-CD115	1:80	BioLegend
APC-conjugated anti-Ly6c	1:10	Miltenyi
FITC-conjugated anti-CD68	1:200	BioLegend
FITC-conjugated anti-CD80	1:50	BioLegend
PE-conjugated anti-CD86	1:20	BioLegend
PE-conjugated anti-CD206	1:40	BioLegend
PE Vio 770-conjugated anti-F4/80	1:10	Miltenyi

### Histology

For PB staining cells were fixed in 4% PFA for 15 min at room temperature. After washing, cells were incubated for 30 min with staining solution (1:1 2% hydrochloric acid (HCl), 2% potassium ferrocyanide [K_4_Fe(CN)_6_]). Following PB exposure cells were rinsed with distilled water and coverslipped with Aqua Poly/Mount (Polysciences Inc., Warrington, PA, USA).

PB staining of tissue sections was conducted with initial incubation for 10 min with 10% K_4_Fe(CN)_6_ followed by 30 min incubation with staining solution (1:1 4% HCl, 4% [K_4_Fe(CN)_6_]). The tissue sections were washed for 5 minutes with tap water, followed by counterstaining of cell nuclei with nuclear fast red for 10 seconds.

For immunohistochemical (IHC) stainings of tissue sections mice were deeply anesthetized with isoflurane and transcardially perfused with 2xPBS followed by 4% PFA. Brains were dissected from skull and post-fixed overnight, before transfer into 30% sucrose solution. Once sunk to the vial’s bottom, brains were frozen in -60°C cold 2-methylbutane and stored at -80°C. 14 μm thin tissue sections of the graft area and 20 μm thin tissue sections of the ischemic lesion site, respectively, were cut in coronal plane using a cryostat (Leica, Wetzlar, Germany) and stored at -20°C.

For immunocytochemistry (ICC), cells were fixed with 4% PFA for 10 min at room temperature. In Table 2 antibodies used for ICC/ IHC are given. For both, IHC and ICC, a nuclei stain was performed (Hoechst Dye 1:1000; Hoechst).

**Table 2:** List of primary (1°) and secondary (2°) antibodies used for ICC and IHC, with corresponding dilutions and distributers.

	Antibody	Host	Dilution ICC/ IHC	Provider
1°	α-Tubulin	rabbit	1:500/---	GeneTex
	anti-dextran FITC	mouse	1:25/---	Stemcell
	luciferase	mouse	---/ 1:50	Santa Cruz
	Iba1	rabbit	---/ 1:1000	Wako
2°	Cy3-anti mouse	donkey	---/ 1:200	Jackson Immuno Research
	Cy5-anti rabbit	donkey	1:200/---	Jackson Immuno Research
	DyLight488-anti rabbit	goat	---/ 1:1000	Pierce
	Hoechst Dye	-----	1:1000/ 1:1000	Hoechst

All stainings of cell and tissue sections were microscopically analysed. For brightfield (BF), phase contrast (PC), as well as fluorescence images of cells and tissue sections, a fluorescent microscope (Keyence BZ-9000) was used with 4x, 20x, 40x, and 60x magnification. Fluorescence 3D-stack of tissue sections was acquired using a confocal microscope (Leica TCS SP8). Here, 20x magnification was chosen. The recorded images were preprocessed using the manufacturers’ software (BZ-II Analyzer 2.1 for the Keyence microscope, Las X Software for confocal microscope). Brightness and contrast were finally adjusted for each experimental setup using ImageJ software (Version 1.48g).

### Statistics

All data are presented as mean ± standard deviation. Comparison of means of two independent groups was conducted using Mann-Whitney test for non parametric data distribution. Multiple comparisons were conducted using either one way ANOVA with Tukey’s post-test (for normal data distribution), or Kruskal-Wallis test followed by Dunn’s correction for non parametric data (Graph Pad Prism version 6.01, GraphPad Software, Inc., San Diego, USA). Longitudinal *in vivo* BLI data was analysed using a repeated measures ANOVA with Bonferroni correction (SPSS version 22, IBM SPSS statistics, Ehningen, Germany). If not specified differently, a p-value ≤ 0.05 was considered significant and highlighted by */^#^, in addition a p ≤ 0.005 was marked by ** and p ≤ 0.001 is represented by ***/^###^.

## Results

### Effectiveness of contrast agent uptake in vitro

**Fig 1:**
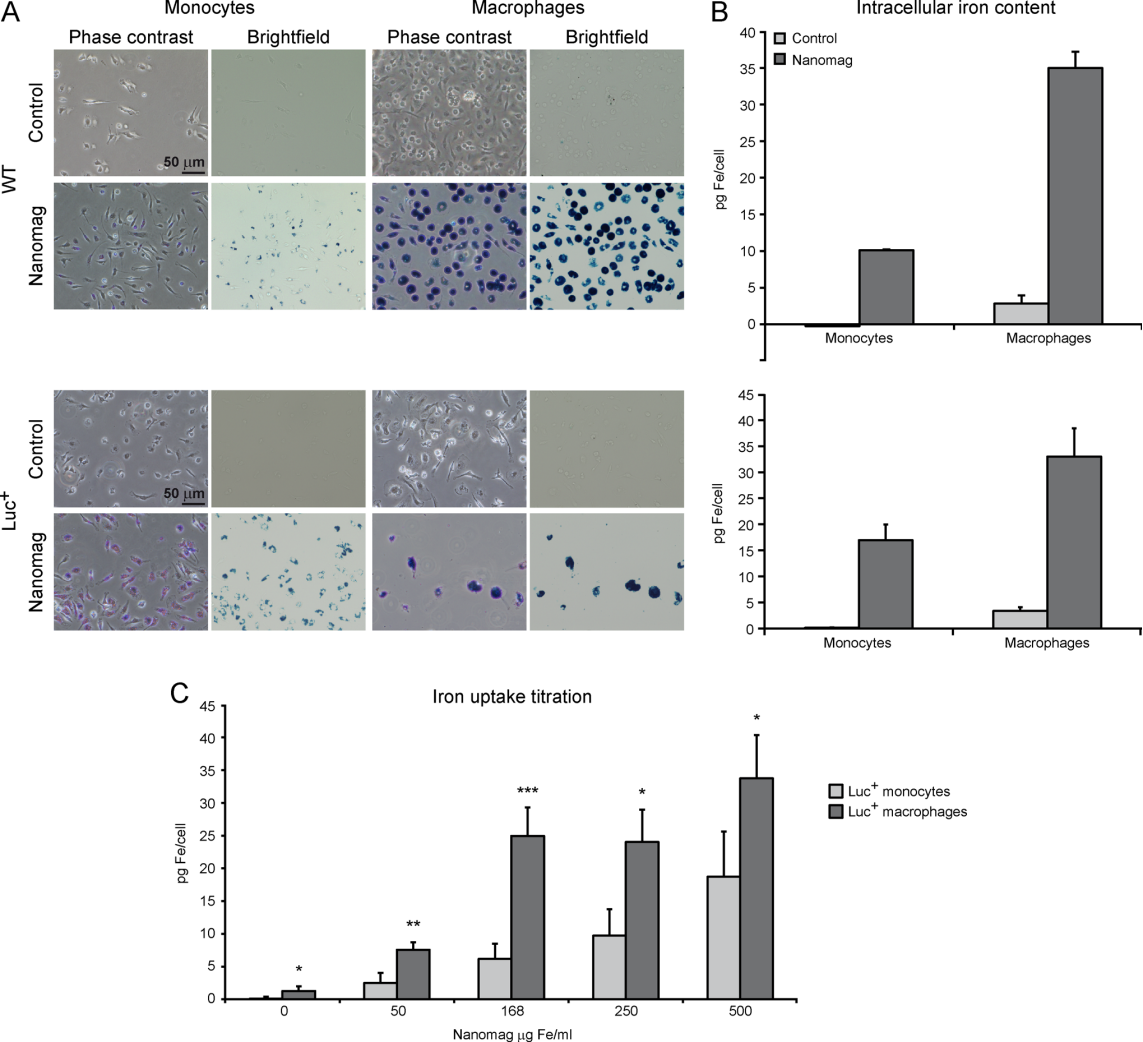
*In vitro* iron labelling efficiency of monocytes and MΦ (WT and luc^+^). (A) Overnight incubation with 168 μg Fe/ml resulted in blue deposits in phase contrast and brightfield microscopy images of PB staining in monocytes and MΦ (scale = 50 μm). (B) Photometric iron content analysis for control (light grey) and contrast agent labelled WT and luc^+^ cells (dark grey; 168 μg Fe/ml), with 2–6 experiments per cell type and Nanomag concentration. (C) Photometric iron uptake titration of luc^+^ monocytes and MΦ. Mean photometric values are displayed as pg Fe/cell. Statistical significance (ANOVA/Kruskal-Wallis: * p ≤ 0.05, ** p ≤ 0.005, *** p ≤ 0.001)always indicates the difference in iron uptake between luc^+^ monocytes and luc^+^ macrophages. Luc^+^ monocytes: 4–11 experiments per concentration, luc^+^ Mɸ: 5–7 experiments per concentration.

Isolated monocytes and differentiated MΦ of C57BL/6 WT and of transgenic β–actin luc mice, as well as MΦ of an immortalized cell line J774A.1, were incubated overnight with Nanomag SPIO particles at a concentration of 168 μg Fe/ml, without the addition of lipofectant agents. Intracellular iron uptake by the cells was clearly visualized by distinct blue deposits after PB staining in phase contrast as well as brightfield microscopy (Fig 1A; S1A Fig), whereas unlabelled control cells remained unstained. Also, ICC staining of control and labelled WT monocytes against the dextran surface coating of the iron oxide particles resulted in clear visualization of the contrast agent particles within the labelled cells (S2 Fig). In contrast, unlabelled control cells did not show fluorescence signal from anti-dextran antibody.

After incubation with 168 μg Fe/ml photometric iron content analysis revealed an iron uptake of 10.12 ± 0.01 pg iron per cell for WT monocytes and 34.96 ± 2.25 pg for WT MΦ (Fig 1B). Intracellular iron content for luc^+^ cells did not differ significantly from WT cells (Mann-Whitney test; monocytes p = 0.13, MΦ p = 0.68), with 16.95 ± 3.02 pg iron/cell for monocytes and 33.00 ± 5.43 pg iron/cell for MΦ.

To investigate iron uptake capacity of luc^+^ monocytes, luc^+^ MΦ and J774A.1 MΦ, photometric analysis was conducted for different iron concentrations (0, 50, 168, 250 and 500 μg Fe/ml). For luc^+^ monocytes, iron uptake continuously increased with rising Nanomag concentrations (Fig 1C), resulting in an iron content of 18.74 ± 6.88 pg Fe/cell for 500 μg Fe/ml. In contrast, iron uptake of luc^+^ MΦ initially peaks at 168 μg Fe/ml with 24.95 ± 4.33 pg Fe/cell and shows a further increase at 500 μg Fe/ml with 33.74 ± 6.661 pg Fe/cell. Iron uptake of the immortalized MΦ cell line J774A.1 reaches a saturation state at 250 μg Fe/ml with 24.06 ± 8.76 pg Fe/cell (S1B Fig). For all tested concentrations, iron content of luc^+^ MΦ was significantly higher compared to luc^+^ monocytes (0, 250 and 500 μg Fe/ml p ≤ 0.05, 50 μg Fe/ml p = 0.002, 168 μg Fe/ml p < 0.001). The immortalized J774A.1 Mɸ only showed significantly higher iron contents for the working concentration of 168 μg Fe/ml (p < 0.001) and 250 μg Fe/ml (p = 0.02). In addition, J774A.1 Mɸ revealed significantly lower intracellular iron contents for 0 μg (p < 0.001), as well as 168 μg Fe/ml (p = 0.03) compared to luc^+^ MΦ (S1B Fig).

### Detection limit of contrast agent labelling in vitro

**Fig 2:**
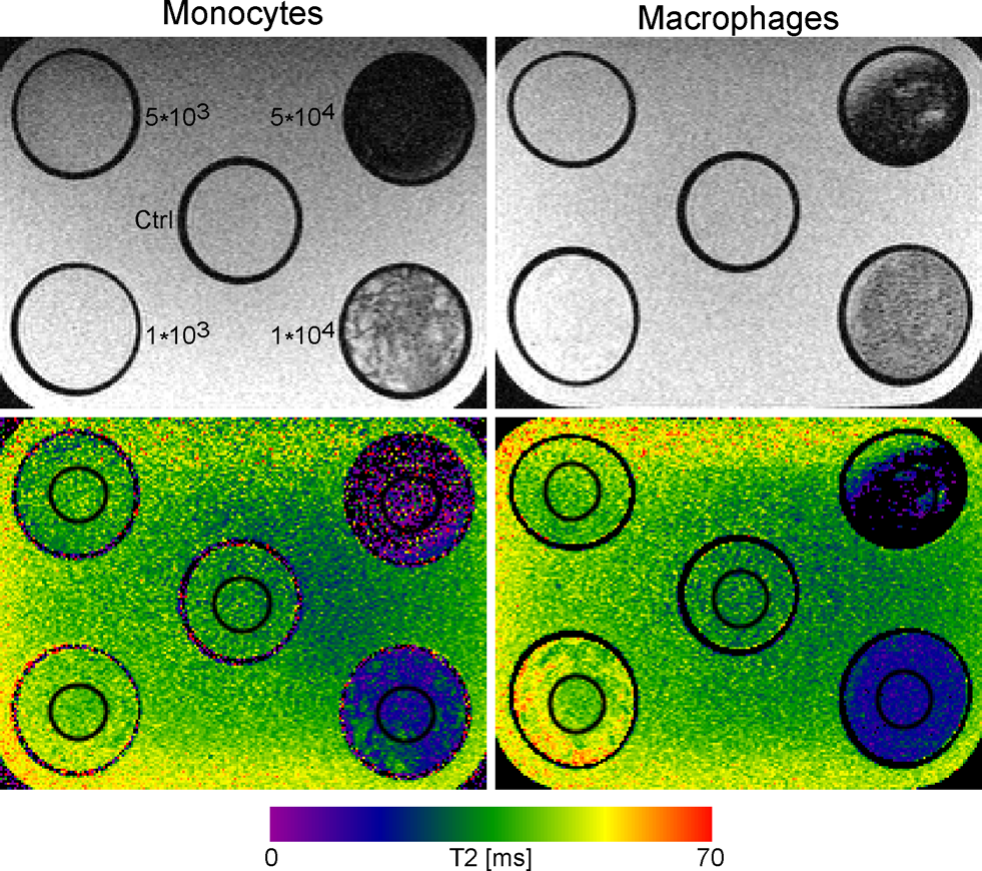
*In vitro* detectability of SPIO labelled monocytes and MΦ (WT). T2-weighted MR images at 11.7 T (TE/TR = 11.25/5,000 ms) of control cells (unlabelled), and of 1x10^3^, 5x10^3^, 1x10^4^ and 5x10^4^ labelled cells (168 μg Fe/ml; upper row) with corresponding quantitative T2 maps (lower row). Next to the probes in the upper left image corresponding cell numbers are noted. The cell numbers also apply for phantoms loaded with MΦ. Images were acquired with a MSME sequence. T2 values (ms) are displayed in the color scale bar below the images.

Determination of cell detection limit and sensitivity *in vitro* with MRI was conducted scanning phantoms loaded with different numbers of SPIO-labelled WT monocytes and MΦ (1x10^3^, 5x10^3^, 1x10^4^, 5x10^4^) (Fig 2). Phantoms loaded with 1x10^4^ and 5x10^4^ labelled cells resulted in clear hypointense signals and a dramatic decrease in T2 relaxation times (monocytes = 19.31 ± 6.45 ms and 6.38 ± 12.21 ms; MΦ = 17.17 ± 4.01 ms and 8.04 ± 9.5 ms) compared to unlabelled controls (monocytes = 33.94 ± 5.53 ms; MΦ = 32.24 ± 5.09 ms). The detection limit *in vitro* was between 5x10^3^ and 1x10^4^ cells for both cell types.

**Fig 3:**
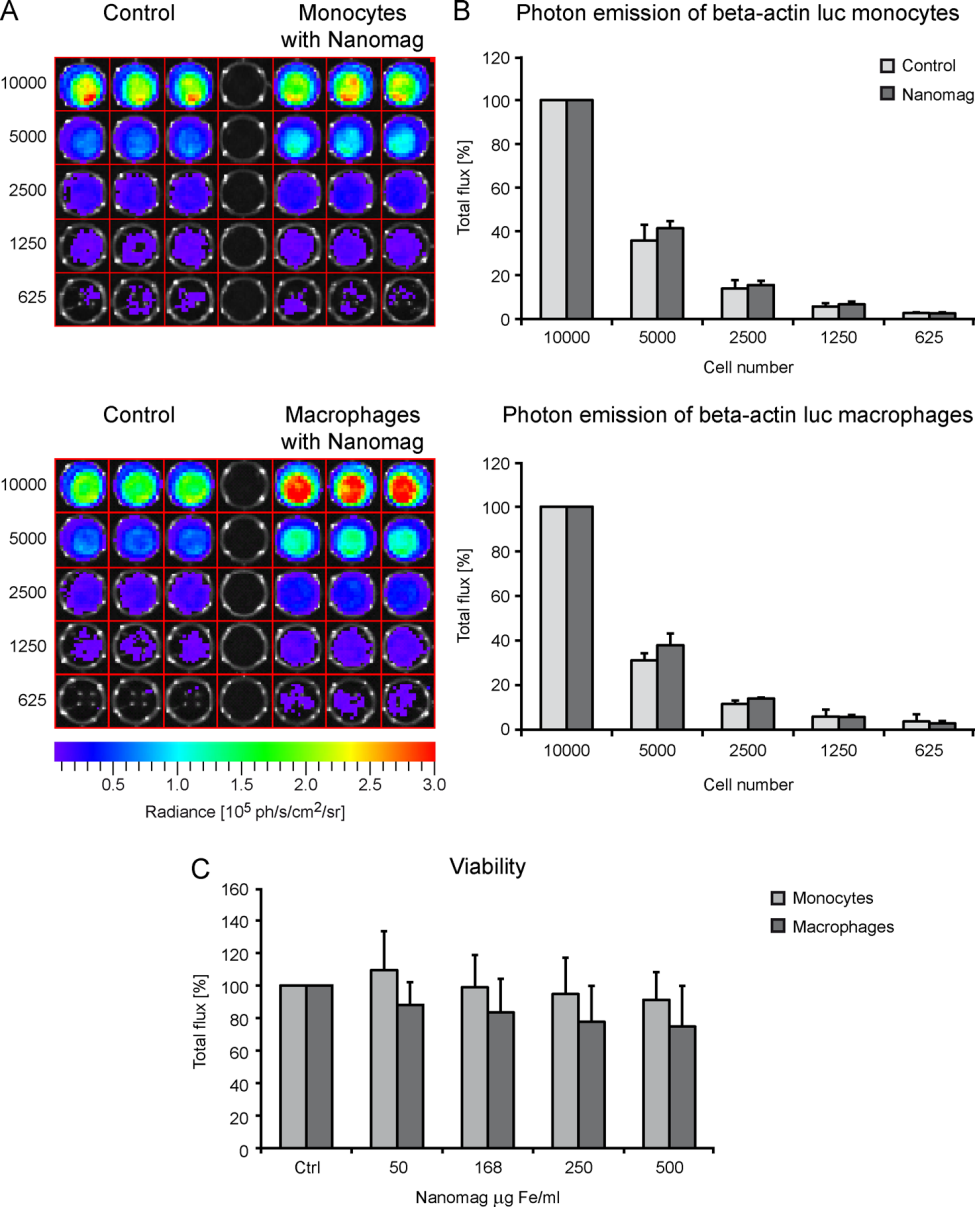
Effect of iron labelling on cell viability. (A) Representative BLI images of an *in vitro* BLI dilution series with unlabelled (control) and SPIO labelled (168 μg Fe/ml) luc^+^ monocytes (upper images) and MΦ (lower images). Values (ph/s/cm^2^/sr) are indicated in the color scale bars below the images. (B) Quantitative plot of mean total flux (ph/s). Values are normalized to photon flux of either 1x10^4^ control (light grey) or labelled (dark grey) cells (for both cell types n = 2, including 3 samples for each cell number). (C) Viability of luc^+^ monocytes (light grey) and MΦ (dark grey) was estimated via Presto Blue reaction for different SPIO concentrations. Values (relative light units–RLUs) were normalized to unlabelled control cells and are displayed as percentage for both cell types (n = 3, with 3 samples for each concentration).

In order to investigate the influence of SPIO-labelling on cell BLI detectability *in vitro*, dilution series of luc^+^ monocytes and luc^+^ MΦ were performed measuring the photon emission of different numbers of control and labelled cells (1x10^4^, 5x10^3^, 2.5x10^3^, 1.25x10^3^, 625) with BLI. Values were averaged over independent experiments, and samples were normalized to photon emission of 1x10^4^ cells. For both cell types, the labelling procedure did not severely alter the photon emission compared to unlabelled control cells for all tested cell amounts. As the enzymatic reaction for the photon emission is energy-dependent, the results testify no dramatic influence of contrast agent labelling on cell viability. For monocytes, a clearly visible signal could still be detected for 2.5x10^3^ control, as well as SPIO-labelled cells (Fig 3A). For MΦ, reliable signals could only be detected for 5x10^3^ or more control and labelled cells. Mean photon emission of the delineated ROIs is plotted in the graphs in Fig 3B.

Cell viability was also calculated for different iron concentrations (Fig 3C) measuring relative light units (RLUs) of the color change of Presto Blue. Viability of both cell types, luc^+^ monocytes and luc^+^ MΦ, decreased slightly with increasing iron concentration, though not significantly (Kruskal-Wallis test: luc^+^ monocytes: p > 0.99 all tested concentrations, luc^+^ MΦ: p > 0.99 for 50 and 168 μg Fe/ml, 250 μg Fe/ml p = 0.48, 500 μg Fe/ml p = 0.68).

### Effect of contrast agent uptake on cell functionality

**Fig 4:**
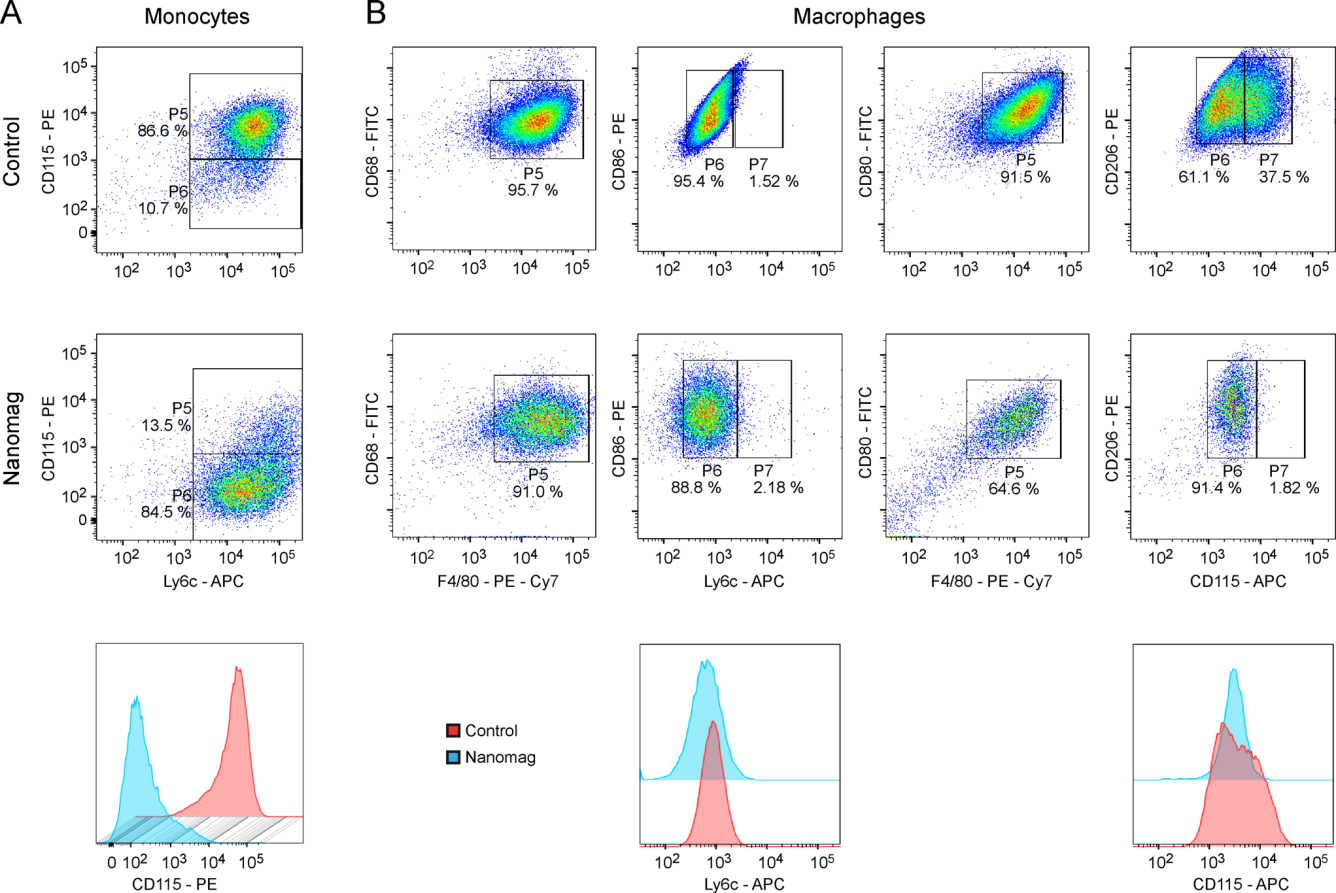
Representative dot plots and histograms of flow cytometric investigations of the effect of SPIO labelling on cell functionality. (A) Unlabelled control (upper row) and Nanomag labelled luc^+^ monocytes (middle row) were stained against monocytic markers Ly6c and CD115. Upon iron particle labelling, monocytes reduced their expression levels for CD115, which is clearly visible in the depicted histogram for CD115 marker (lower row). (B) Luc^+^ MΦ without and with SPIO-labelling were tested for their expression levels of CD68, CD80, CD86, CD206 and F4/80. The monocytic markers Ly6c and CD115 served as negative controls. For both, labelled and unlabelled MΦ, F4/80-positive cells were also positive for CD68, CD80, CD86 and CD206. No change in expression levels of Ly6c could be detected upon particle uptake, whereas the CD115^+^ population of control cells minimized due to contrast agent incorporation. n = 2 for both cell types.

Influence of SPIO-labelling on functionality of luc^+^ monocytes and differentiated luc^+^ MΦ was analysed via flow cytometric measurements. Labelled (168 μg Fe/ml) as well as unlabelled control monocytes were stained for the monocytic markers CD115 and Ly6c (Fig 4A). 86.6% of the unlabelled control cells were double positive for both tested markers. Only a small population (10.7%) did not express CD115. This situation changed upon contrast agent uptake. Only 13.5% of the cells were double positive, but 84.5% reduced their CD115-expression and only expressed Ly6c.

Unlabelled and labelled luc^+^ MΦ were stained for the common MΦ marker F4/80, the active phagocytosis marker CD68, the markers for antigen presentation on fully functional MΦ CD80/CD86 and CD206, another marker for phagocytosis of pathogens, as well as antigen-presentation and resolution of inflammation. As negative control, cells were stained against Ly6c and the monocytic marker CD115 (Fig 4B). Labelled as well as unlabelled macrophages were first gated for positive F4/80-expression (gate P5), as shown in the plots of the first or third column of Fig 4B. In a second step, these F4/80^+^ populations were gated for further marker expressions, as depicted in gates P6 and P7 in the plots of the second and fourth column of Fig 4B. These plots show that F4/80-positive control MΦ as well as labelled MΦ did also express CD68, CD80, CD86 and CD206. Nanomag labelling resulted in two populations for CD115 marker, a low and a high one. The positive CD115 population for control cells disappeared almost entirely upon iron particle uptake. In contrast, expression levels for Ly6c did not change noticeably upon Nanomag incorporation.

### In vivo detectability of transplanted cells

**Fig 5:**
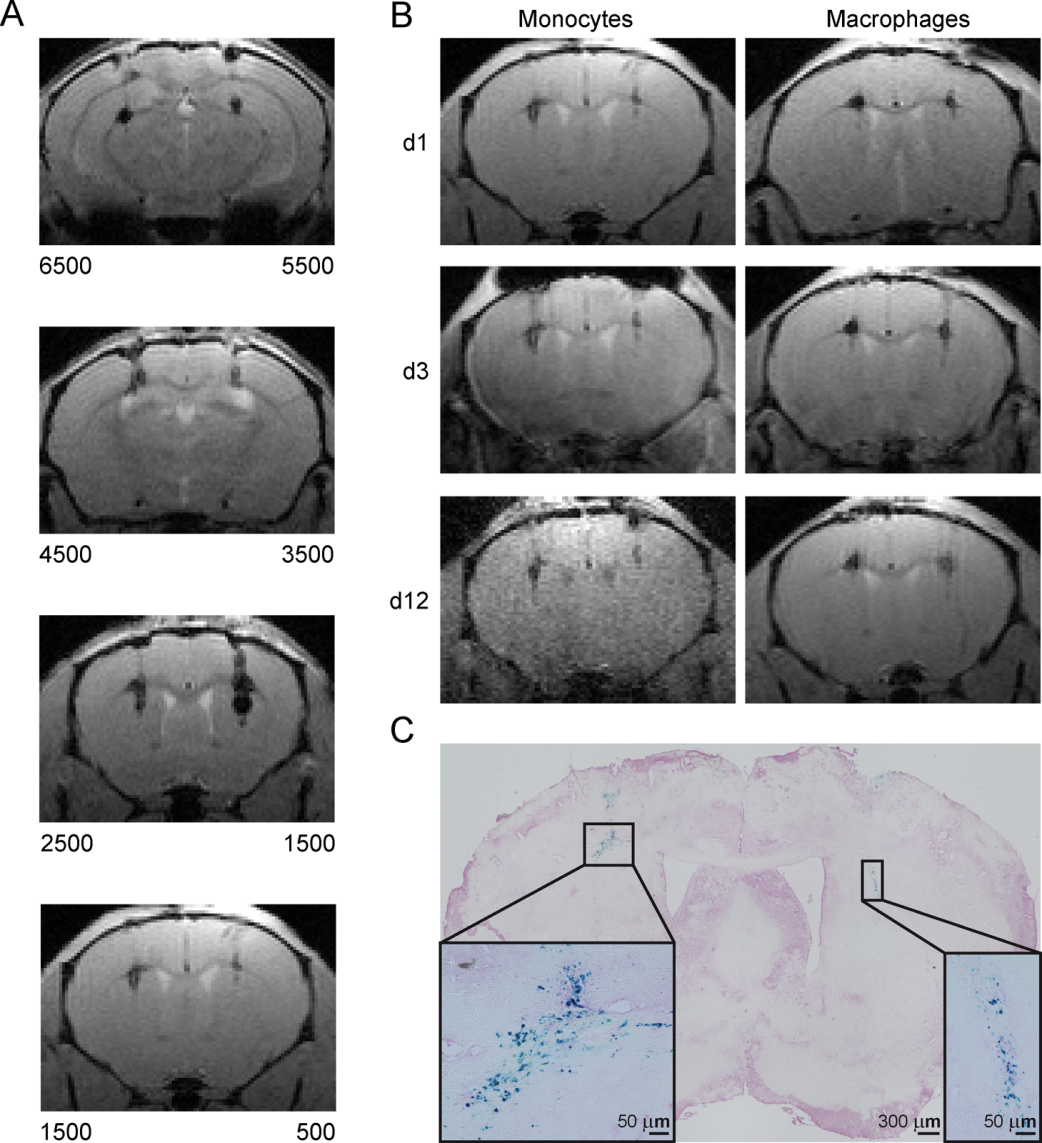
*In vivo* longitudinal detectability of grafted monocytes and MΦ using MRI at 9.4 T. (A) Qualitative MR images (MGE sequence) of graft titration with different numbers of SPIO labelled monocytes (WT), with the cell numbers indicated below both hemispheres, n = 1. Transplanted cells are visible as small hypointense clusters. (B) Imaging time course of transplanted WT monocytes and MΦ for days 1, 3 and 12 post transplantation. Left hemisphere 1500, right hemisphere 500 cells, n = 1 per cell type. (C) Representative PB staining of brain tissue section indicating grafted monocytes as PB^+^ (blue) cells. Scale = 50 μm.

Detection limit was determined conducting an *in vivo* transplantation titration series. Different numbers of Nanomag-labelled WT monocytes (6.5x10^3^, 5.5x10^3^, 4.5x10^3^, 3.5x10^3^, 2.5x10^3^, 1.5x10^3^ and 500; all 168 μg Fe/ml) were stereotactically transplanted into the brain of mice at a depth of 3.0 mm and an AP distance of 0.5 mm. T2*-weighted MR images were acquired for visualization of cell grafts (Fig 5A). As few as 500 grafted cells produced clear hypointense spots on MR images. T2* relaxation times for the different cell numbers did not allow reliable quantification due to substantial signal loss already at smaller cell numbers (S3 Fig). T2*-weighted MRI therefore was only used for image contrast assessments.

Longitudinal observation of graft location of WT monocytes and MΦ was executed with T2*-weighted MRI measurements on day 1, 3 and 12 post cell transplantation. Labelled monocytes and MΦ (168 μg Fe/ml) were transplanted into the striatum of C57BL/6 mice (left hemisphere: 1,500 cells; right hemisphere: 500 cells). Even 12 days after transplantation 500 grafted cells could still be detected. Signal intensity as well as location did not change noticeably for both cell types (Fig 5B). For identification of grafted cells on histological brain sections, PB staining was conducted resulting in PB^+^ areas that were in accordance with hypointense regions on MR images (Fig 5C).

### Survival and fate of grafted monocytes and MΦ—correlation with immunostaining

**Fig 6:**
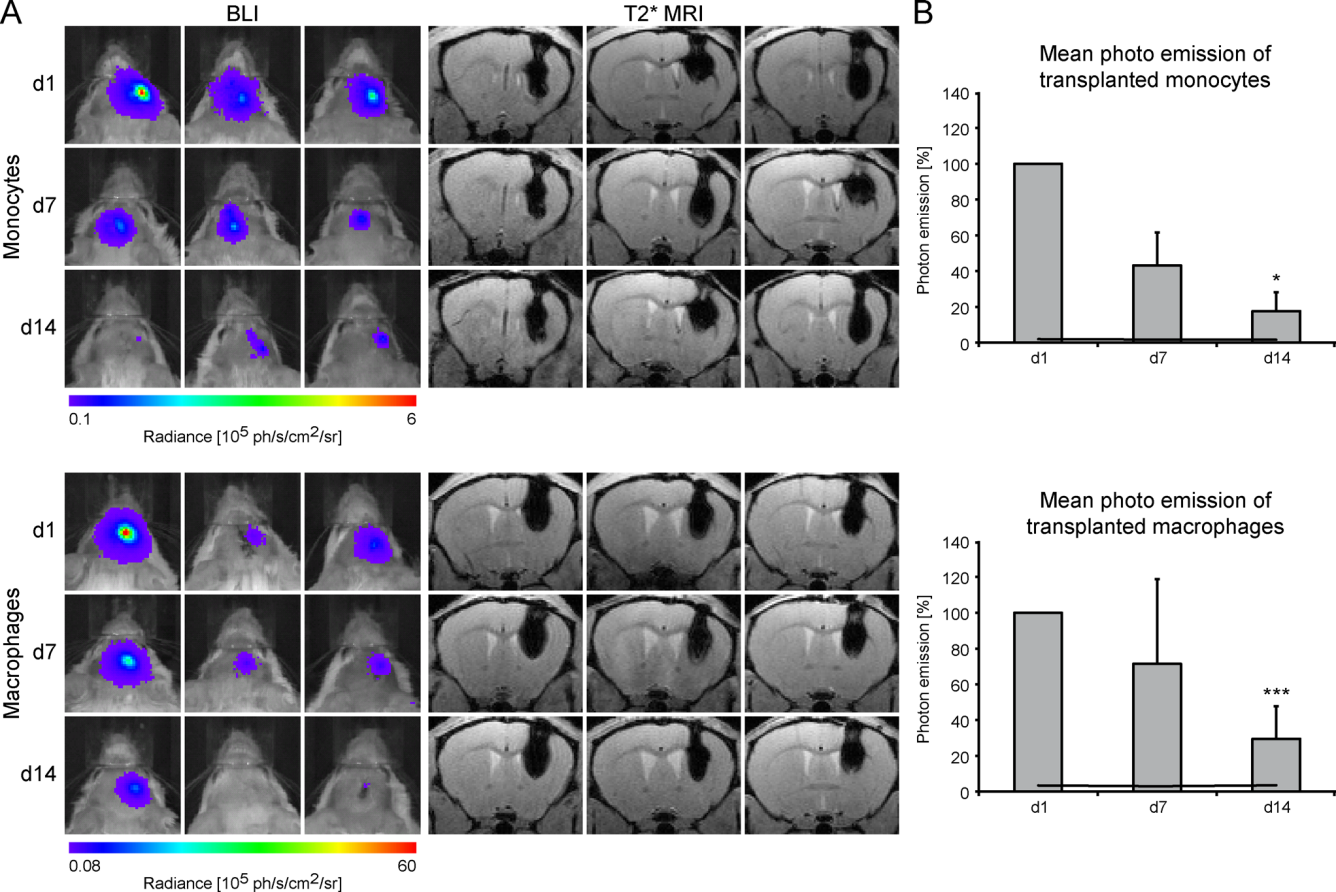
Survival of grafted, SPIO labelled luc^+^ monocytes and MΦ. (A) BLI time course with corresponding qualitative MR images for 3 individual animals per cell type, presented for days 1, 7 and 14 post transplantation into the striatum. MR images were acquired at 11.7 T using a T2*-weighted MGE sequence and serve for verification of graft location. Values of photon emission are indicated in the color scale bar below the images. (B) Corresponding mean photon emission of the 3 individual animals for the three time points. Values are normalized to d1 post transplantation and plotted as percentage. Black lines display mean background signal of the animals. Statistical significance indicated by * and *** apply for comparison with photon emission on day 1.

We further validated the longitudinal survival of grafted cells. For this purpose, 7.5x10^4^ luc^+^ monocytes and luc^+^ MΦ labelled with 168 μg Fe/ml were transplanted into the striatum of the right hemisphere of 3 animals per cell type. Repetitive BLI measurements on days 1, 7 and 14 served as viability surveillance, whereas MRI was conducted for temporal observation of migratory activity of transplanted cells (Fig 6A). Photon emission of both, luc^+^ monocytes and luc^+^ MΦ decreased in all animals over time. After 14 days, BLI signals were significantly reduced compared to photon emission on day 1 (monocytes (F(2,4) = 49.717, p = 0.016); MΦ (F(2,10) = 26.536, p < 0.001)). In contrast, hypointense regions representing the cell grafts only slightly decreased in intensity while no clear migration was observed. 14 days after transplantation, 30 ± 18% of the initial BLI signal was detectable for luc^+^ MΦ and 18 ± 10% for luc^+^ monocytes (Fig 6B).

Prussian Blue combined with nuclear fast red staining revealed localization of iron mostly within cells and not in parenchyma (S4A Fig). On IHC sections stained for luciferase and Iba1, double positive cells in the graft area could be clearly visualized. Migration and recruitment behavior of endogenous, luc^-^ microglia and MΦ was observed with a 3D stack acquired at the confocal microscope. Intriguingly, a clear accumulation of these cells (Iba1^+^, green) around the cell graft containing luc^+^/Iba1^+^ monocytes/MΦ was visible (S4B Fig).

### Systemic application of monocytes and macrophages following cerebral ischemia

Experimental ischemic stroke was induced in eight mice and 24 hours later, either monocytes or MΦ were injected through the tail vein to monitor by imaging the time profile of recruitment to the ischemic lesion. Sequential T_2_*-weighted MRI on day 2, 3 and 4 post ischemia was performed for complete observation during the early acute phase after stroke.

**Fig 7:**
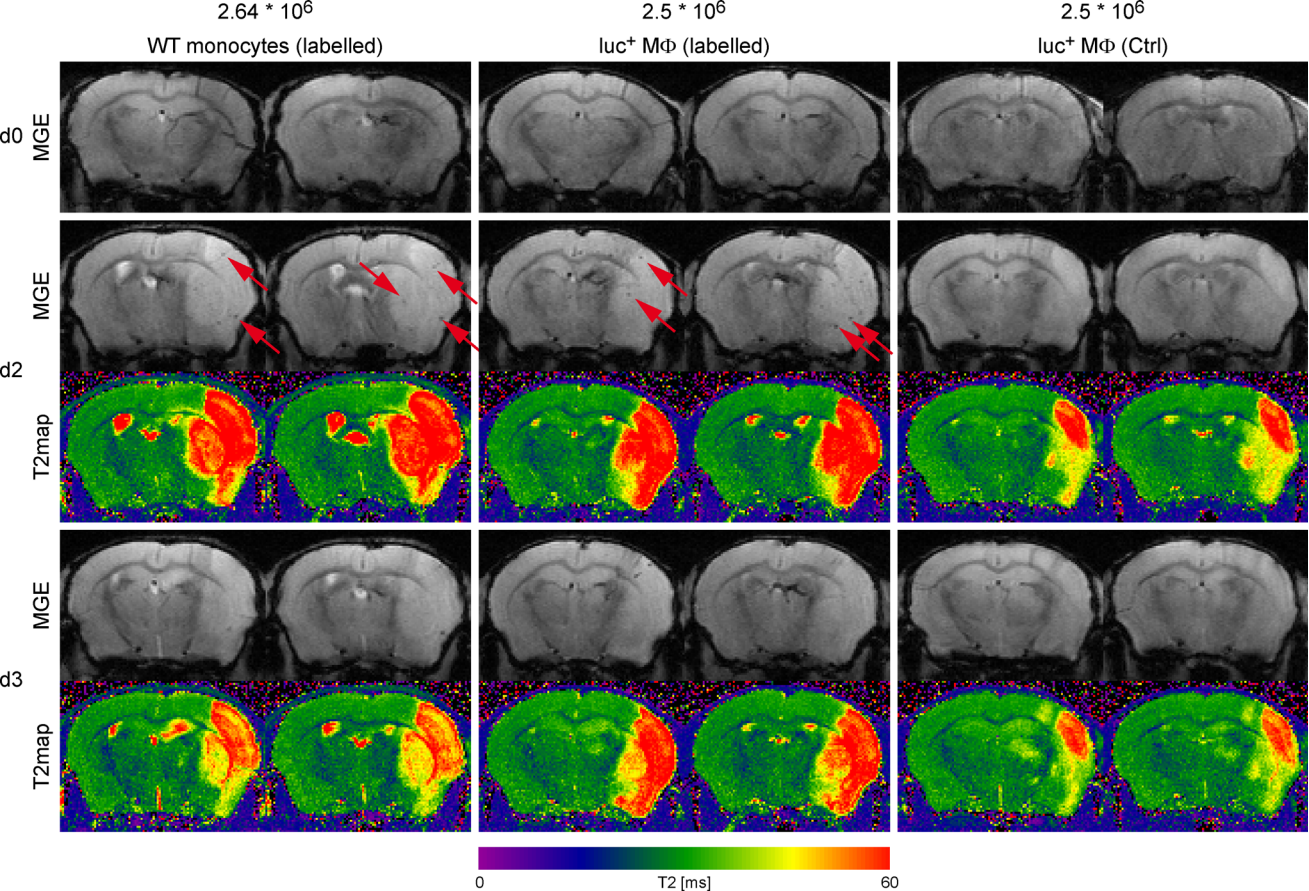
Representative MR images of i.v. injected WT monocytes and luc^+^ MΦ. Displayed are T2*-weighted images (second echo; TE = 8.5ms), acquired with a MGE sequence at 11.7T directly after MCAO surgery and before cell injection (upper row, d0), on day 2 as well as day 3 post stroke (1 and 2 days post cell injection) with corresponding T2 maps calculated for better stroke visualization. T2 relaxation times are displayed with the color scale bar below the images. Left column: contrast agent labelled WT monocytes (2.64x10^6^); center column: contrast agent labelled luc^+^ MΦ (2.5x10^6^); right column: unlabelled control luc^+^ MΦ (2.5x10^6^). Hypointense signal voids evoked by cells reaching the ischemic brain territory are indicated by red arrows.

Two days after stroke (one day after cell injection), small hypointense spots of labelled WT monocytes as well as labelled luc^+^ MΦ could be detected on T_2_*-weighted images, mostly on the ischemic hemisphere (Fig 7, left and center columns) as highlighted by the red arrows. Comparable hypointense signals could not be observed before cell injection (d0) and were not visible when injecting unlabelled control cells (Fig 7, right column). However, the observed signal voids vanished by day 3 post stroke for both cell types.

Animals injected with luc^+^ MΦ underwent additional subsequent BLI measurements on day 2 and 3 post stroke. Despite the pronounced MRI signal on day 2, BLI measurement at the same time point resulted in diffuse photon emission mostly arising from areas in the thorax (S5 Fig). On histological tissue sections acquired 4 days post MCAO, the hypointense signal voids detected with MRI could not be precisely co-localized with accumulated PB^+^ areas. However, scattered PB^+^ cells were clearly visible on brain sections for both, injected WT monocytes, as well as luc^+^ MΦ (data not shown).

## Discussion

We have successfully established a highly efficient labelling protocol, enabling the investigation and characterisation of monocyte and MΦ transplants with multimodal imaging techniques *in vivo*. Moreover, we show that the efficient labelling strategy based on concatenation of the use of luciferase-expressing transgenic cells and physical labelling with iron oxide nanoparticles was successfully established *in vitro* and validated for the influence on cell functionality. SPIO labelling facilitates cell tracking with MRI and generates information on spatial localization. The complementary optical imaging of luciferase expression provides insight in the functional fate of the transplanted cells. In addition, the transgene also serves as target for antibody staining on IHC tissue sections at the end of experiments.

Titration of the provided iron concentration revealed higher iron uptake with increasing iron concentrations for all tested cell types, but reaching a saturation state for luc^+^ MΦ at 168 μg Fe/ml and for the immortalized J774A.1 MΦ line at 250 μg Fe/ml. However, intracellular iron content of luc^+^ monocytes and J774A.1 MΦ was remarkably lower than for luc^+^ MΦ, possibly resulting in the saturation at higher iron concentration for J774A.1 MΦ and no detectable saturation even at the highest tested concentration for luc^+^ monocytes. These findings lead to the decision of using the working concentration of 168 μg Fe/ml for all *in vivo* experiments. Also, the chosen concentration of iron oxide nanoparticles resulted in very effective labelling of virtually every cell, as visible on PB microscopic images. In addition, luc^+^ MΦ exhibited significantly higher iron storage for the working concentration compared to J774A.1 MΦ. This highlights the benefits of newly differentiated primary cells over commercially available immortalized MΦ (S1B Fig).

Of utmost importance for any longitudinal study, the labelling process with contrast agent did not significantly alter cell viability, as could be demonstrated with Presto Blue staining of luc^+^ monocytes and MΦ and seen in the comparable BLI signal intensity. On the contrary, for monocytes the viability level remained comparable to unlabelled control cells, even increased slightly for an iron concentration of 50 μg Fe/ml. Viability of luc^+^ MΦ continuously decreased with increasing iron concentration, whereas measured photon emission of Nanomag labelled cells was slightly higher in some cases compared to control cells for *in vitro* BLI. This phenomenon was previously reported by De Vocht and colleagues [22]. They could show that iron particles needed to be located intracellularly in order to increase *in vitro* BLI signal in viable cells and in cell lysates. Their explanation for this phenomenon is that particle uptake might influence the ionic composition of the cytoplasm, resulting in increased stability/activity of the luciferase protein. This explanation could also support the fact that the observed BLI amplification is not as pronounced for luc^+^ monocytes, as their intracellular iron content for the working concentration of 168 μg Fe/ml is approximately three times lower compared to luc^+^ MΦ. Hence, the proposed stabilization/activation effect on luciferase would not be as pronounced.

Furthermore, flow cytometric analysis of cell functionality demonstrated only little changes upon contrast agent uptake. Interestingly, for both cell types, luc^+^ monocytes and luc^+^ MΦ expression levels of the monocytic marker CD115 were lower for labelled cells compared to unlabelled control cells. Although CD115 is predominantly expressed by monocytes, MΦ are also known to express the marker [23–25]. We believe that this shift in expression levels upon contrast agent uptake may be due to an incomplete differentiation of several cells within the population, resulting in different maturity stages. In any case, this CD115^+^ population disappeared almost entirely upon iron particle uptake for both cell types, indicating completion of differentiation in the case of MΦ and potentially starting differentiation in the case of monocytes.

We were able to define with MRI phantoms that the *in vitro* threshold of detection was between 1x10^3^ and 5x10^3^ cells. In contrast, with our highly efficient labelling protocol we could clearly detect small cell clusters of as few as 500 transplanted cells *in vivo*, which resulted in hypointense signal spots on T2*-weighted MR images for up to 12 days post transplantation. The difference of a factor of 10 in the detection limits is explained with the different volumes that the cells are dissolved in. For *in vitro* MRI, the cells are prepared in a total volume of 30 μl, whereas for *in vivo* transplantation a total volume of 2 μl is injected. Hence, the cells are more densely located in a MRI voxel for *in vivo* experiments, than in the phantom.

MRI is the gold standard method for cell tracking, due to the fact that it is non-invasive and provides high spatial resolution, and is therefore eligible for repetitive measurements on the same animal. The use of luciferase-expressing transgenic cells in combination with optical imaging offers information on cell viability. Completion of the two methods enables a longitudinal, repetitive and multimodal observation of transplanted cells in the living animal. After transplantation of luc^+^ monocytes and luc^+^ MΦ into the striatum of recipient mice, we were able to detect the BLI signal from viable cells for up to 2 weeks post transplantation for both cell types. The average signal on day 14 represented about 18% of the original signal for monocytes and 30% for MΦ. Although the detected signal lies above the background signal of the animals, we observed inter-individual differences resulting in high error bars. One reason for these variations is the i.p. administration method of luciferin chosen for BLI. Though all measurements were conducted by the same person, slight injection variations can never be completely avoided, leading to altered distribution time profiles of the substrate and differences in photon emission. A second source for errors might lie in the cell injection procedure itself. When retracting the syringe too fast, chances are high that part of the cell suspension is drawn up through the injection canal. Additionally, despite proper resuspension of the cell suspension before transplantation, iron loaded cells are quite heavy and quickly sink to the bottom of the Hamilton syringe. Thus, an equal number of cells for all animals cannot be guaranteed. In addition, luc^+^ MΦ seem to have a slightly better survival rate during the first week after transplantation. One possible explanation could be the target tissue: upon traumatic injuries and other cerebral diseases monocytes enter the brain from blood vessels and, by doing so, differentiate into tissue MΦ. However, direct transplantation of the cells without any recruitment signals to trigger their differentiation might hinder their tissue integration and lead to faster cell death compared to already differentiated MΦ.

Interestingly, despite decreasing photon emission over time, hypointense signal voids on MR images remained relatively unchanged in spatial localization as well as contrast. This is in line with recent studies, reporting long term persistence of SPIO generated signals from transplanted, GFP expressing BV-2 microglia for up to 90 days [26]. With combined Prussian Blue and IHC staining for Iba1, Cianciaruso and colleagues monitored a co-localization of PB^+^ areas with Iba1^+^ but GFP^-^ cells. These cells showed a ramified morphology, typical for endogenous microglia and/or infiltrating MΦ [27–29]. Another group reported similar persistence of signal after death of SPIO labelled cells [15]. From our PB tissue staining, we cannot exclude iron particle uptake by endogenous and/or infiltrating phagocytic cells. Nevertheless, released SPIOs accumulating in brain parenchyma is unlikely, as most of the blue deposits are co-localized to cell nuclei. Acquisition of a 3D stack of IHC brain sections of mice transplanted with luc^+^ MΦ clearly visualised enclosure of cell graft by endogenous Iba1^+^ cells. Similar spatial distribution patterns were documented for spinal cord injuries, where recruited MΦ located at the margins of the lesion and activated microglia were found at the border regions as well as within the lesion centre [7].

The results of the translational approach indicate that the suggested method needs to be optimized for systemic *in vivo* application in an animal model of stroke. We were able to detect clear hypointense spots for labelled cells that were not visible when injecting unlabelled control cells. However, an observation time of up to 5 weeks as reported by Stroh and colleagues [30] could not be reproduced. One possible explanation could be the isolation of spleen-derived monocytes and the systemic administration of these cells after contrast agent labelling in splenectomised recipient mice in order to avoid homing of the cells to their host organ.

A general comment about different detection thresholds appears appropriate at this point. The above discussed changes in detection thresholds for controlled in vitro and in vivo conditions get even more complicated when including the challenge of observing the small cell clusters in our pathophysiological conditions (cf discussion below). It emphasizes the difficulty in designing in vitro and in vivo situations reflecting the relevant in vivo pathological situation for truely translational studies.

While MRI allows to detect a clear signal in the brain from the labelled macrophages at day 2, BLI measurement of animals injected with luc^+^ MΦ at the same time point was not successful. This is explained by the small numbers of cells in individual locations (represented by the discrete small signal voids in MRI). The BLI signal originating from such small cell clusters or even individual cells deep in the brain is absorbed by the surrounding tissue, thus inhibiting successful BLI measurement. Despite the large number (2.5x10^6^) of injected monocytes or macrophages we have been able to detect only a few discrete spots on the ischemic territory and only for a short time window after injection. Recent quantitative immunohistochemical analysis of i.v. injected 4x10^6^ GFP^+^ monocytes resulted in only approximately 6.000 cells in the stroke area [31]. Considering this minute fraction of injected cells successfully infiltrating the brain, our own findings emphasise the high sensitivity and the extraordinary potential of the presented strategy where we could clearly detect hypointense signal voids on MR images.

A clear limitation of our present study is the lack of unambiguous discrimination of the location of the labelled cells in the brain after systemic injection. It remains to be investigated whether all the cells detected on MRI on day 2 post injection have entered the parenchyma or whether some of them remained in the perivascular or intravascular space. Future extensive histological analysis will have to address this point.

Our presented highly efficient *in vitro* labelling protocol provides a good basis and step stone to investigate cellular processes of inflammation in a multimodal approach. We expect that with further developments, especially continuous improvement of sensitivity to allow following small numbers of cells over several days, the in vivo imaging will facilitate future investigations of the important and distinct role of tissue-infiltrating monocyte-derived MΦ in regulating neuroinflammation on the one hand and the impact of this cell type on neuronal regeneration after stroke on the other hand.

## Supporting Information

S1 Fig *In vitro* iron uptake capacity. (A) PB staining of PFA-fixated control (top row) and labelled (168 μg Fe/ml; bottom row) J774A.1 Mɸ. Microscopy images were acquired with phase contrast and brightfield. Intracellular iron storage was clearly visible as bright blue deposits. Scale = 50 μm. (B) Intracellular iron content after incubation with different contrast agent concentrations. Values are plotted as pg iron per cell. Statistical significance indicated by * (ANOVA/Kruskal-Wallis: * p ≤ 0.05, *** p ≤ 0.001) refers to differences in iron uptake by luc^+^ monocytes of the same Nanomag concentration, as were presented in Fig 1C. Statistical significance ^#^ (ANOVA/Kruskal-Wallis: ^#^ p ≤ 0.05, ^###^ p ≤ 0.001) describes differences in iron uptake by luc^+^ Mɸ of the same iron particle concentration (Fig 1C). n = 3 with at least 10 samples per concentration.
(TIF)

S2 Fig ICC staining of unlabelled control (left) and contrast agent labelled (right) WT monocytes. Counterstaining against dextran surface coating of the contrast agent (green) visualizes high uptake and dense storage of the particles by almost every cell. This staining is missing for unlabelled cells. Microtubules were stained with α-tubulin (red), cell nuclei were stained with Hoechst dye (blue). Scale control cells = 30 μm, labelled cells = 20 μm. n = 1.
(TIF)

S3 Fig T2* relaxation times for transplanted WT monocytes. Cells were incubated overnight with 168 μg Fe/ml Nanomag particles, and different cell numbers were stereotactically transplanted into brain tissue of 1 C57BL/6 WT recipient mouse. T2* was was recorded at 9.4T and calculated in ROIs of T2* maps on graft locations, T2* relaxation times (ms) were calculated and plotted for each cell number.
(TIF)

S4 Fig Representative histology and immunohistological staining of grafted luc^+^ Mɸ. (A) Overview of graft location (scale = 300 μm) and close-up of area indicated by black box (scale = 50 μm). Incorporated SPIO particles could be identified as blue deposits in PB staining. To visualize cell nuclei, tissue sections were also stained with nuclear fast red. (B) Fluorescence microscopy images of grafted Mɸ. To distinguish transplants from brain residing microglia and endogenous tissue infiltrating Mɸ, brain sections were double stained against luciferase (red) and Iba1 (green). Grafted luc^+^ Mɸ could be identified as double positive (orange) cells in the overlay 3D stack acquired on a confocal microscope. Endogenous Iba1^+^ cells (green) recruited to transplantation site enclose grafted cells. Time point 14 days post transplantation. Scale = 100 μm.
(TIF)

S5 Fig BLI signal of Mɸ after systemic application. BLI signal in mice after systemic injection with unlabelled and Nanomag labelled luc^+^ Mɸ was measured on day 3 post MCAO (2 days post i.v. injection). Images were acquired for whole body or head only. For the latter the body was covered with black cardboard in order to collect photon emission from the heads only. Values of emitted photons are indicated in the color scale bar below the images.
(TIF)
