# Supplementary figures and images for: In Vivo Non-Invasive Tracking of Macrophage Recruitment to Experimental Stroke
Source: PLoS One. 2016 Jun 24;11(6):e0156626. doi: 10.1371/journal.pone.0156626 (PMC4920382; doi:10.1371/journal.pone.0156626)

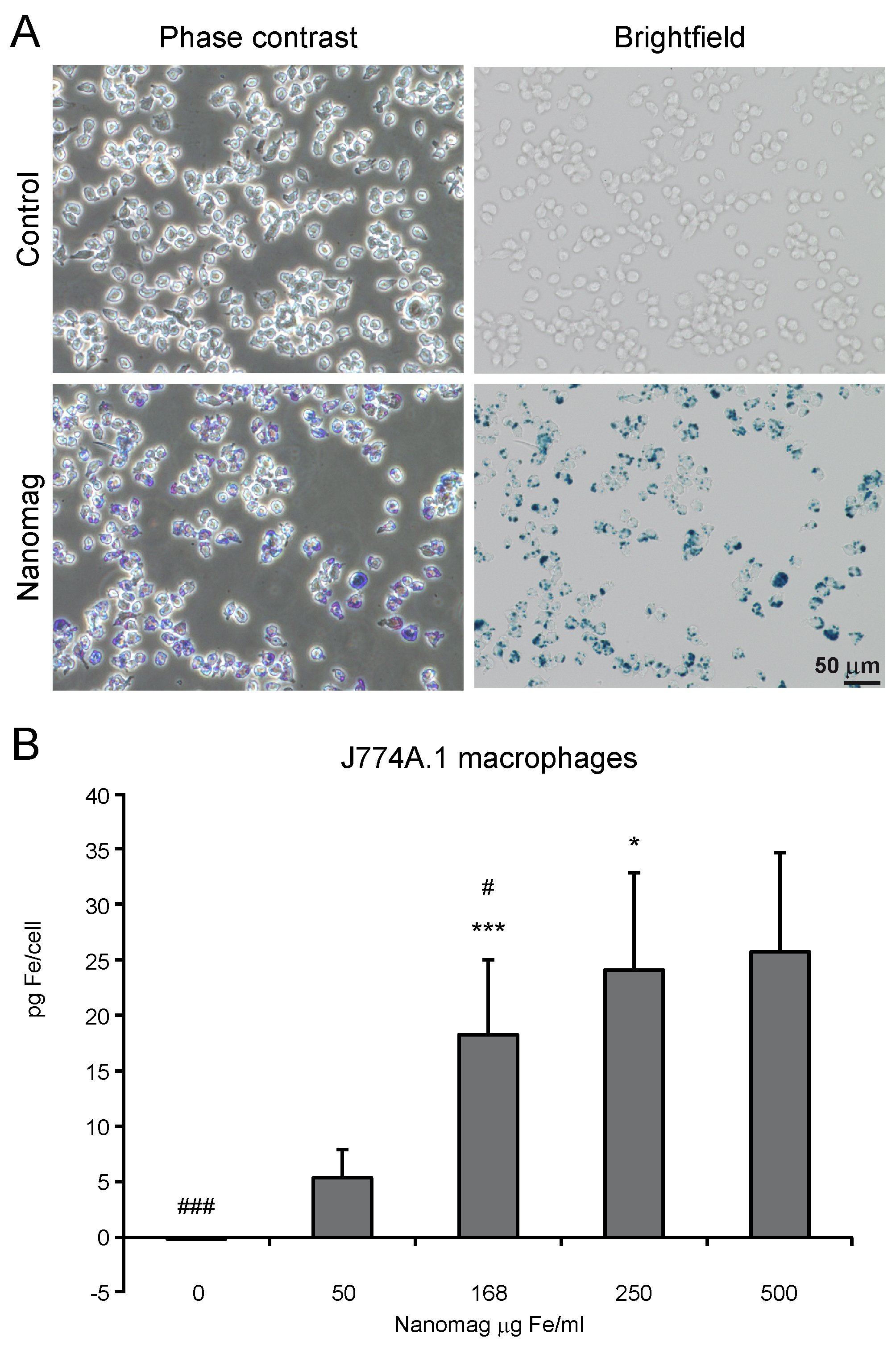

Supplement: S1 Fig — (A) PB staining of PFA-fixated control (top row) and labelled (168 μg Fe/ml; bottom row) J774A.1 Mɸ. Microscopy images were acquired with phase contrast and brightfield. Intracellular iron storage was clearly visible as bright blue deposits. Scale = 50 μm. (B) Intracellular iron content after incubation with different contrast agent concentrations. Values are plotted as pg iron per cell. Statistical significance indicated by * (ANOVA/Kruskal-Wallis: * p ≤ 0.05, *** p ≤ 0.001) refers to differences in iron uptake by luc+ monocytes of the same Nanomag concentration, as were presented in Fig 1C. Statistical significance # (ANOVA/Kruskal-Wallis: # p ≤ 0.05, ### p ≤ 0.001) describes differences in iron uptake by luc+ Mɸ of the same iron particle concentration (Fig 1C). n = 3 with at least 10 samples per concentration. (TIF) [file pone.0156626.s001.tif]

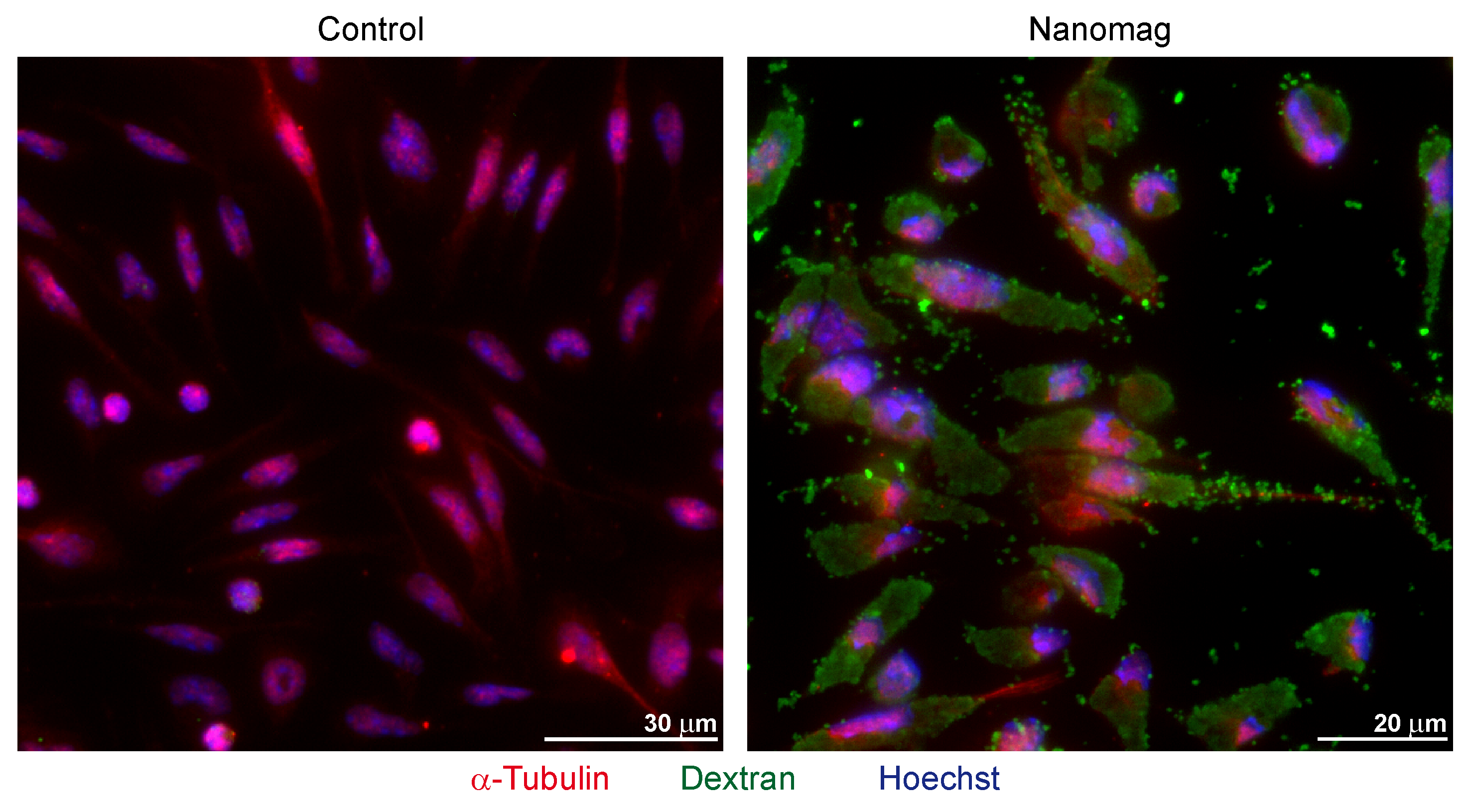

Supplement: S2 Fig — Counterstaining against dextran surface coating of the contrast agent (green) visualizes high uptake and dense storage of the particles by almost every cell. This staining is missing for unlabelled cells. Microtubules were stained with α-tubulin (red), cell nuclei were stained with Hoechst dye (blue). Scale control cells = 30 μm, labelled cells = 20 μm. n = 1. (TIF) [file pone.0156626.s002.tif]

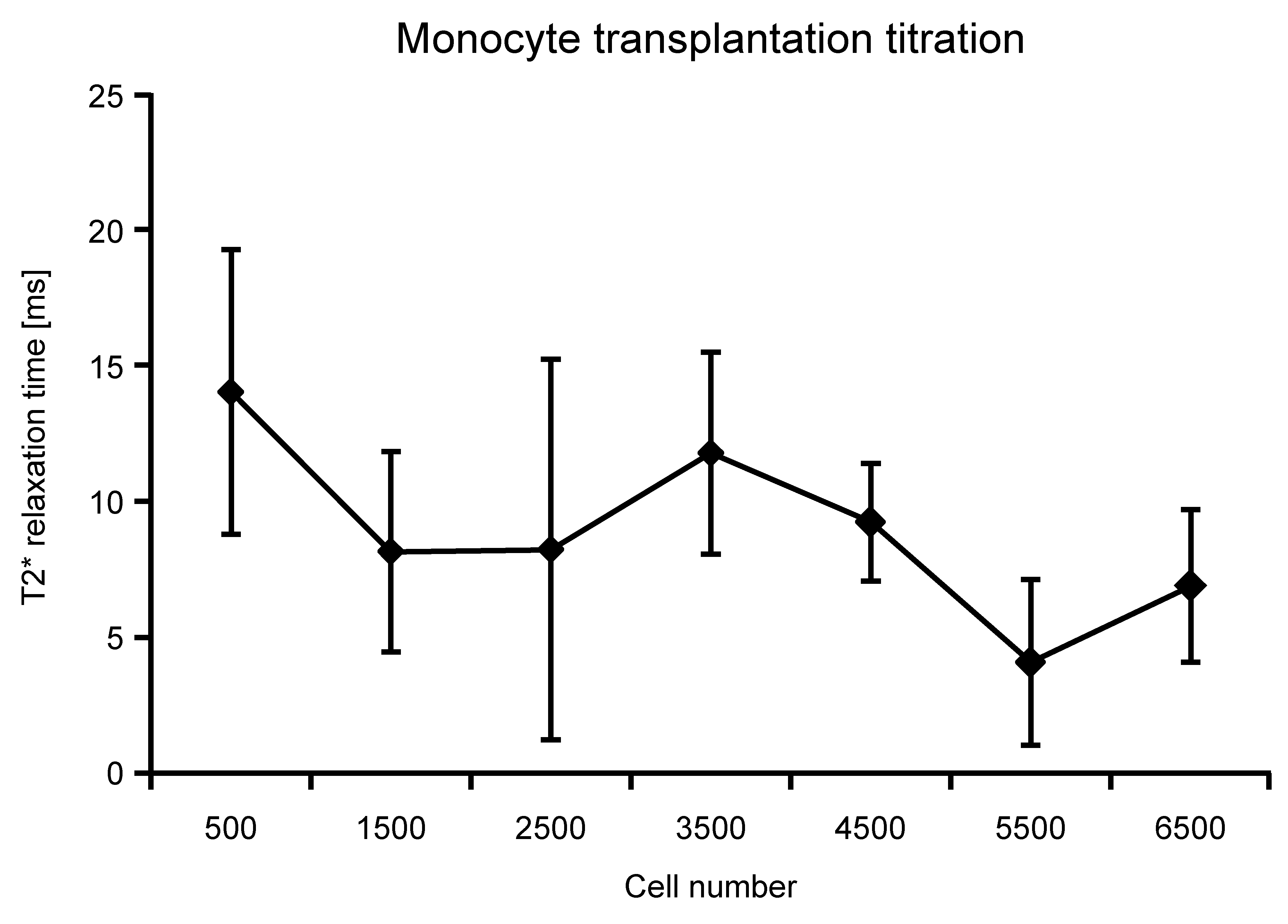

Supplement: S3 Fig — Cells were incubated overnight with 168 μg Fe/ml Nanomag particles, and different cell numbers were stereotactically transplanted into brain tissue of 1 C57BL/6 WT recipient mouse. T2* was was recorded at 9.4T and calculated in ROIs of T2* maps on graft locations, T2* relaxation times (ms) were calculated and plotted for each cell number. (TIF) [file pone.0156626.s003.tif]

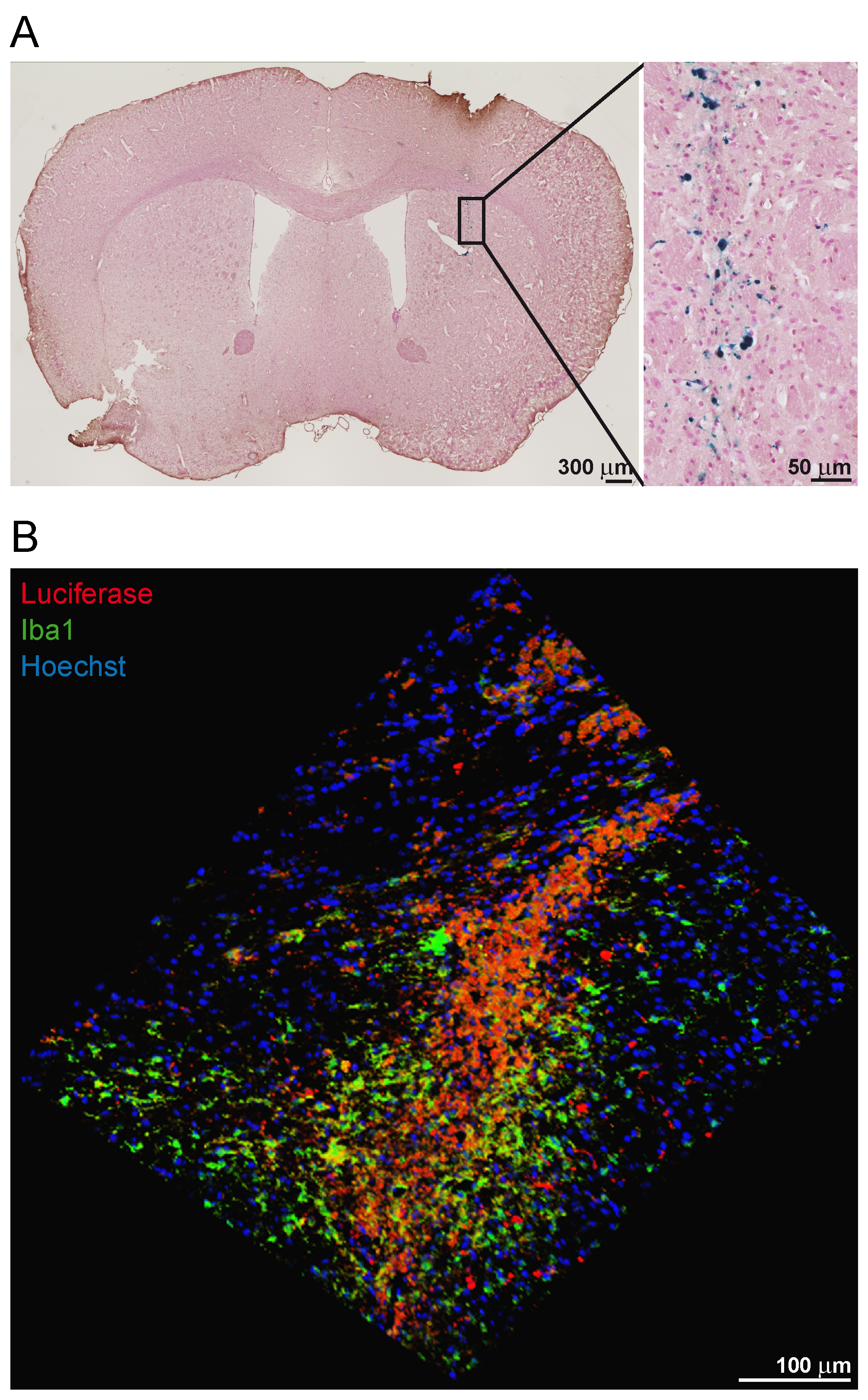

Supplement: S4 Fig — (A) Overview of graft location (scale = 300 μm) and close-up of area indicated by black box (scale = 50 μm). Incorporated SPIO particles could be identified as blue deposits in PB staining. To visualize cell nuclei, tissue sections were also stained with nuclear fast red. (B) Fluorescence microscopy images of grafted Mɸ. To distinguish transplants from brain residing microglia and endogenous tissue infiltrating Mɸ, brain sections were double stained against luciferase (red) and Iba1 (green). Grafted luc+ Mɸ could be identified as double positive (orange) cells in the overlay 3D stack acquired on a confocal microscope. Endogenous Iba1+ cells (green) recruited to transplantation site enclose grafted cells. Time point 14 days post transplantation. Scale = 100 μm. (TIF) [file pone.0156626.s004.tif]

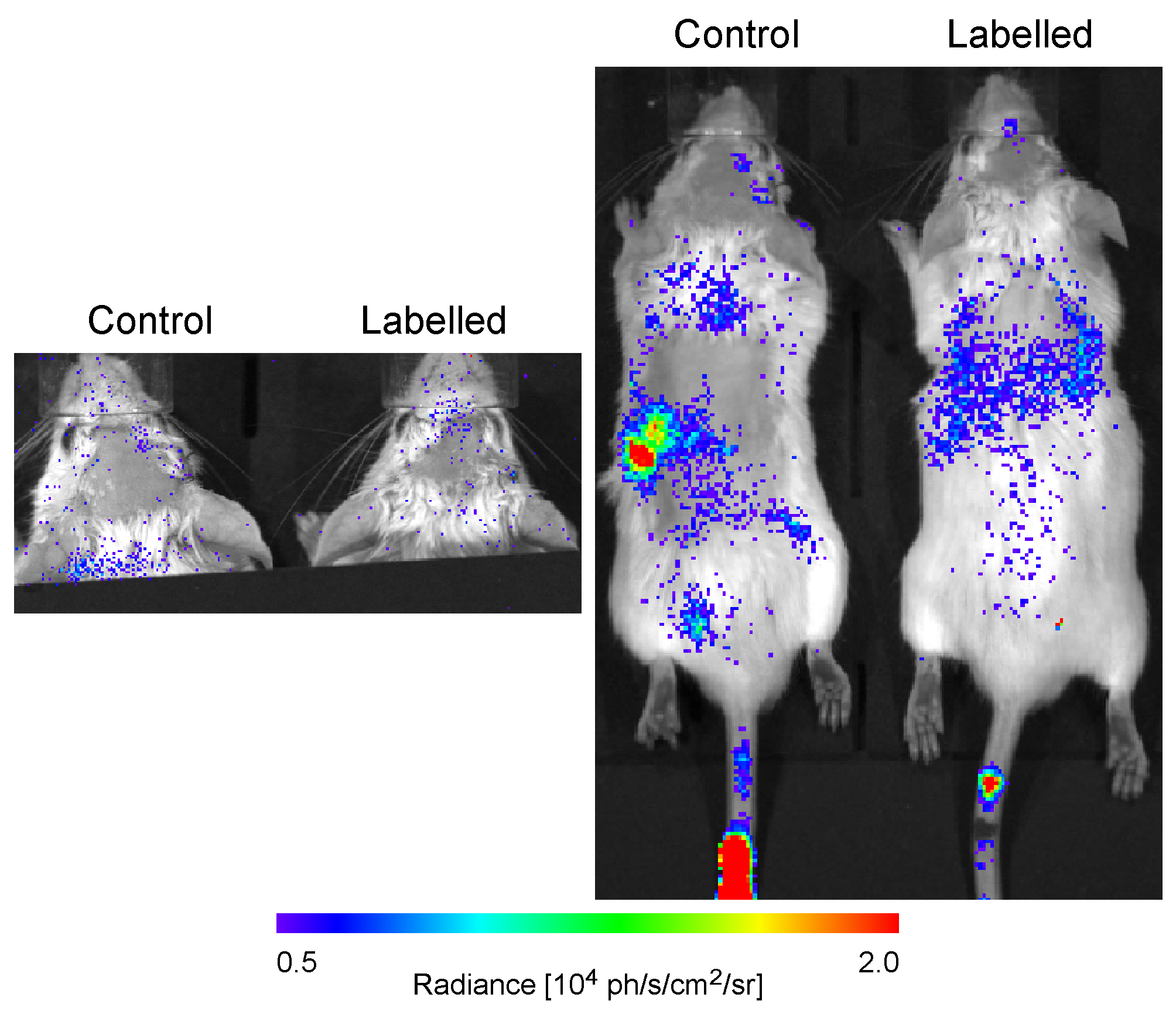

Supplement: S5 Fig — BLI signal in mice after systemic injection with unlabelled and Nanomag labelled luc+ Mɸ was measured on day 3 post MCAO (2 days post i.v. injection). Images were acquired for whole body or head only. For the latter the body was covered with black cardboard in order to collect photon emission from the heads only. Values of emitted photons are indicated in the color scale bar below the images. (TIF) [file pone.0156626.s005.tif]
